# 5-[1-(1,3-Dimethyl-2,4,6-trioxohexa­hydropyrimidin-5-yl)-2-oxoprop­yl]-1,3-di­methyl­pyrimidine-2,4,6(1*H*,3*H*,5*H*)-trione

**DOI:** 10.1107/S1600536813020138

**Published:** 2013-07-27

**Authors:** Kamal Sweidan, Manfred Steimann

**Affiliations:** a Department of Chemistry, The University of Jordan, Amman 11942, Jordan; b Institut für Anorganische Chemie der Universität Tübingen, Auf der Morgenstelle 18, D-72076 Tübingen, Germany

## Abstract

The title compound, C_15_H_18_N_4_O_7_, is a product of the substitution reaction of 5,5-di­bromo-1,3-di­methyl­barbituric acid with sodium sulfide in aqueous acetone. In the crystal, mol­ecules display neither inter­molecular nor intra­molecular hydrogen bonding and the two barbiturate rings adopt the keto form.

## Related literature

For general applications of barbituric acid, see: Negwer (2001 ▶); Bojarski *et al.* (1985 ▶); Sans & Chosaz (1988 ▶). For the structures of related compounds, see: Sweidan *et al.* (2009 ▶); Ahadi *et al.* (2012 ▶). For the synthesis of the starting material, see: Sweidan *et al.* (2010 ▶).
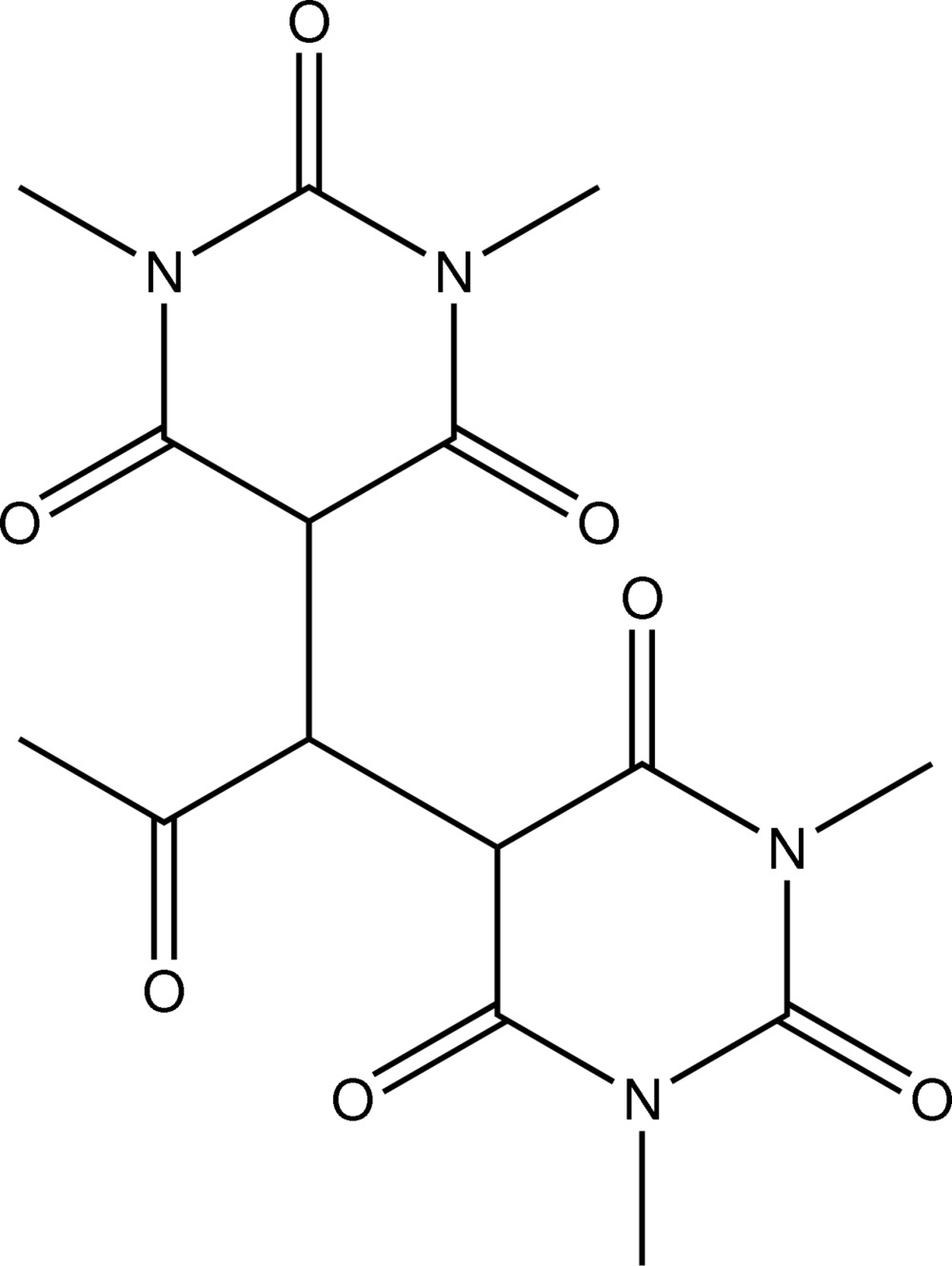


## Experimental

### 

#### Crystal data

C_15_H_18_N_4_O_7_
*M* *_r_* = 366.33
Orthorhombic, 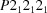

*a* = 9.253 (2) Å
*b* = 13.179 (3) Å
*c* = 13.360 (3) Å
*V* = 1629.2 (6) Å^3^
*Z* = 4
Mo *K*α radiation
μ = 0.12 mm^−1^
*T* = 213 K
0.50 × 0.35 × 0.25 mm

#### Data collection

Enraf–Nonius CAD-4 diffractometer
1911 measured reflections
1911 independent reflections
1265 reflections with *I* > 2σ(*I*)
3 standard reflections every 200 reflections intensity decay: 1.0%

#### Refinement

*R*[*F* ^2^ > 2σ(*F* ^2^)] = 0.043
*wR*(*F* ^2^) = 0.099
*S* = 1.07
1911 reflections
240 parameters
H-atom parameters constrained
Δρ_max_ = 0.17 e Å^−3^
Δρ_min_ = −0.17 e Å^−3^

### 

Data collection: *CAD-4 Software* (Enraf–Nonius, 1998 ▶); cell refinement: *SET4* and *CEKDIM* in *CAD-4 Software*; data reduction: *HELENA*/*PLATON* (Spek, 2009 ▶); program(s) used to solve structure: *SHELXS97* (Sheldrick, 2008 ▶); program(s) used to refine structure: *SHELXL97* (Sheldrick, 2008 ▶); molecular graphics: *SHELXTL* (Sheldrick, 2008 ▶); software used to prepare material for publication: *SHELXTL*.

## Supplementary Material

Crystal structure: contains datablock(s) global, I. DOI: 10.1107/S1600536813020138/mw2109sup1.cif

Structure factors: contains datablock(s) I. DOI: 10.1107/S1600536813020138/mw2109Isup2.hkl

Supplementary material file. DOI: 10.1107/S1600536813020138/mw2109Isup3.cml

Additional supplementary materials:  crystallographic information; 3D view; checkCIF report

## Figures and Tables

**Fig. 1.**
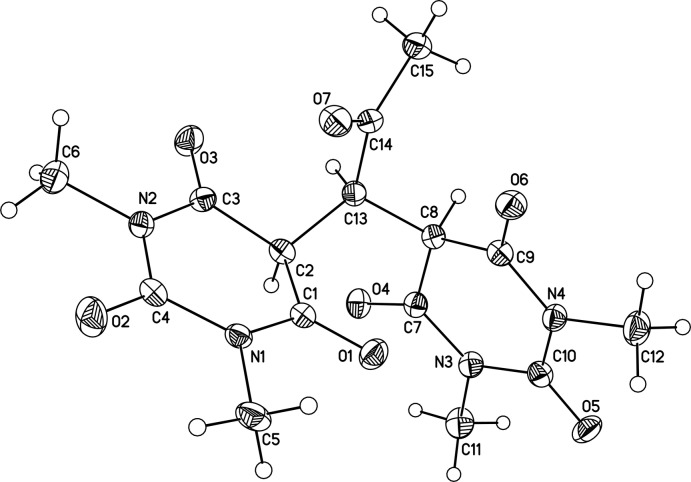
The molecular structure of the title molecule showing the atom numbering scheme with 20% probability displacement ellipsoids for non-H atoms.

## supplementary crystallographic information

### Comment

Many 1,3-dimethylbarbituric acid derivatives play important roles in the areas of pharmaceutical and medicinal chemistry (Bojarski *et al.*, 1985; Sans & Chosaz, 1988). In the target molecule, two moieties of the 1,3-dimethylbarbiturate anion were attached to the same carbon of the acetone molecule and hydrogen sulfide and sodium bromide were produced during the course of the reaction. It is clear that each barbiturate ring adopts a keto form rather than the enol form as indicated by the bond angles C1-C2-C3 (116.1 (3)°), C3-C2-C13 (109.1 (2)°) and C1-C2-C13 (115.5 (2)°) for one barbiturate ring and C7-C8-C13 (112.6 (3)°), C9-C8-C13 (114.4 (3)°) and C7-C8-C9 (116.31 (3)°) for the second one. In addition, the bond lengths C12-C13 (153.7 (4) pm) and C13-C8 (154.3 (4) pm) lie within the normal range for a carbon-carbon (sp^3^-sp^3^) single bond length. Due to the steric bulk of the barbiturate rings, the bond angle C2-C13-C8 (116.2 (2)°) is noticeably larger than C2-C13-C14 (110.4 (3)°) or C8-C13-C14 (113.0 (3)°).

### Experimental

The title compound, C_15_H_18_N_4_O_7_, was prepared by addition of a solution of 5,5-dibromo-1,3-dimethylbarbituric acid (Sweidan *et al.*, 2010), (0.74g, 2.4 mmol) in 10 mL of acetone to a solution of sodium sulfide (0.19g, 2.4 mmol) in 15 mL of water at room temperature. After the reaction mixture was stirred overnight, the precipitate was filtered off and dried in vacuo. The yield after recrystallisation from dichloromethane/diethyl ether was 0.24g (22%) as colorless crystals.

### Refinement

Hydrogen atoms were included in the refinement at calculated positions C—H = 0.95–1.00 Å and with *U*_iso_(H) = 1.2*U*_eq_(aromatic C) or 1.5*U*_eq_(aliphatic C), using a riding-model approximation.

### Crystal data

**Table:**

C_15_H_18_N_4_O_7_	*F*(000) = 768
*M**_r_* = 366.33	*D*_x_ = 1.494 Mg m^−^^3^
Orthorhombic, *P*2_1_2_1_2_1_	Mo *K*α radiation, λ = 0.71073 Å
Hall symbol: P 2ac 2ab	Cell parameters from 25 reflections
*a* = 9.253 (2) Å	θ = 6.8–15.5°
*b* = 13.179 (3) Å	µ = 0.12 mm^−^^1^
*c* = 13.360 (3) Å	*T* = 213 K
*V* = 1629.2 (6) Å^3^	Cube, colourless
*Z* = 4	0.50 × 0.35 × 0.25 mm

### Data collection

**Table:**

Enraf–Nonius CAD-4 diffractometer	*R*_int_ = 0.000
Radiation source: fine-focus sealed tube	θ_max_ = 26.4°, θ_min_ = 3.1°
Graphite monochromator	*h* = 0→11
ω scans	*k* = 0→16
1911 measured reflections	*l* = 0→16
1911 independent reflections	3 standard reflections every 200 reflections
1265 reflections with *I* > 2σ(*I*)	intensity decay: 1.0%

### Refinement

**Table:**

Refinement on *F*^2^	Primary atom site location: structure-invariant direct methods
Least-squares matrix: full	Secondary atom site location: difference Fourier map
*R*[*F*^2^ > 2σ(*F*^2^)] = 0.043	Hydrogen site location: inferred from neighbouring sites
*wR*(*F*^2^) = 0.099	H-atom parameters constrained
*S* = 1.07	*w* = 1/[σ^2^(*F*_o_^2^) + (0.0391*P*)^2^ + 0.2519*P*] where *P* = (*F*_o_^2^ + 2*F*_c_^2^)/3
1911 reflections	(Δ/σ)_max_ < 0.001
240 parameters	Δρ_max_ = 0.17 e Å^−^^3^
0 restraints	Δρ_min_ = −0.17 e Å^−^^3^

### Special details

**Table:**

Geometry. All esds (except the esd in the dihedral angle between two l.s. planes) are estimated using the full covariance matrix. The cell esds are taken into account individually in the estimation of esds in distances, angles and torsion angles; correlations between esds in cell parameters are only used when they are defined by crystal symmetry. An approximate (isotropic) treatment of cell esds is used for estimating esds involving l.s. planes.
Refinement. Refinement of F^2^ against ALL reflections. The weighted R-factor wR and goodness of fit S are based on F^2^, conventional R-factors R are based on F, with F set to zero for negative F^2^. The threshold expression of F^2^ > 2sigma(F^2^) is used only for calculating R-factors(gt) etc. and is not relevant to the choice of reflections for refinement. R-factors based on F^2^ are statistically about twice as large as those based on F, and R- factors based on ALL data will be even larger.

### Fractional atomic coordinates and isotropic or equivalent isotropic displacement parameters (Å^2^)

**Table:**

	*x*	*y*	*z*	*U*_iso_*/*U*_eq_	
N1	0.1811 (3)	0.5023 (2)	0.09198 (18)	0.0356 (6)	
N2	0.2638 (3)	0.38060 (19)	−0.02592 (18)	0.0375 (7)	
N3	0.6557 (3)	0.6949 (2)	0.23141 (19)	0.0404 (7)	
N4	0.5211 (3)	0.82117 (18)	0.1472 (2)	0.0397 (7)	
O1	0.3230 (3)	0.62919 (18)	0.14586 (18)	0.0551 (7)	
O2	0.0383 (3)	0.37348 (19)	0.0384 (2)	0.0646 (8)	
O3	0.4834 (3)	0.39452 (18)	−0.09365 (19)	0.0559 (7)	
O4	0.7066 (3)	0.54505 (17)	0.15638 (17)	0.0462 (6)	
O5	0.5992 (3)	0.84280 (18)	0.30569 (17)	0.0576 (7)	
O6	0.4264 (3)	0.79063 (16)	−0.00524 (17)	0.0488 (6)	
O7	0.3341 (3)	0.60948 (19)	−0.12316 (17)	0.0517 (7)	
C1	0.3085 (4)	0.5526 (2)	0.0950 (2)	0.0374 (8)	
C2	0.4327 (3)	0.5085 (2)	0.0391 (2)	0.0347 (8)	
H2	0.4939	0.4763	0.0909	0.042*	
C3	0.3974 (4)	0.4251 (2)	−0.0330 (2)	0.0388 (8)	
C4	0.1547 (4)	0.4153 (2)	0.0347 (2)	0.0382 (8)	
C5	0.0635 (4)	0.5370 (3)	0.1582 (2)	0.0512 (10)	
H5A	0.0940	0.5312	0.2275	0.077*	
H5B	−0.0216	0.4954	0.1474	0.077*	
H5C	0.0408	0.6073	0.1434	0.077*	
C6	0.2332 (4)	0.2914 (3)	−0.0893 (3)	0.0557 (10)	
H6A	0.1466	0.2578	−0.0654	0.084*	
H6B	0.3141	0.2447	−0.0861	0.084*	
H6C	0.2189	0.3131	−0.1579	0.084*	
C7	0.6562 (3)	0.6294 (3)	0.1511 (2)	0.0375 (8)	
C8	0.5953 (3)	0.6702 (2)	0.0545 (2)	0.0335 (7)	
H8	0.6815	0.6938	0.0173	0.040*	
C9	0.5027 (4)	0.7636 (2)	0.0630 (2)	0.0365 (8)	
C10	0.5932 (4)	0.7903 (3)	0.2325 (3)	0.0429 (9)	
C11	0.7255 (4)	0.6633 (3)	0.3251 (2)	0.0573 (11)	
H11A	0.7699	0.5973	0.3159	0.086*	
H11B	0.6537	0.6593	0.3778	0.086*	
H11C	0.7989	0.7124	0.3434	0.086*	
C12	0.4578 (4)	0.9231 (2)	0.1506 (3)	0.0570 (11)	
H12A	0.4153	0.9390	0.0862	0.085*	
H12B	0.5327	0.9722	0.1660	0.085*	
H12C	0.3838	0.9255	0.2019	0.085*	
C13	0.5319 (3)	0.5860 (2)	−0.0127 (2)	0.0359 (8)	
H13	0.6162	0.5464	−0.0364	0.043*	
C14	0.4602 (4)	0.6271 (2)	−0.1071 (2)	0.0410 (8)	
C15	0.5562 (4)	0.6769 (3)	−0.1827 (2)	0.0547 (10)	
H15A	0.6098	0.6254	−0.2189	0.082*	
H15B	0.6232	0.7219	−0.1488	0.082*	
H15C	0.4978	0.7157	−0.2292	0.082*	

### Atomic displacement parameters (Å^2^)

**Table:**

	*U*^11^	*U*^22^	*U*^33^	*U*^12^	*U*^13^	*U*^23^
N1	0.0331 (18)	0.0352 (16)	0.0383 (16)	0.0004 (16)	0.0103 (15)	0.0043 (15)
N2	0.0343 (19)	0.0382 (18)	0.0393 (17)	−0.0073 (16)	−0.0013 (15)	−0.0007 (15)
N3	0.038 (2)	0.0413 (18)	0.0420 (16)	−0.0019 (17)	−0.0140 (17)	0.0002 (16)
N4	0.039 (2)	0.0324 (15)	0.0457 (18)	0.0022 (16)	−0.0040 (17)	−0.0023 (15)
O1	0.0511 (19)	0.0493 (18)	0.0644 (17)	−0.0075 (17)	0.0168 (17)	−0.0184 (16)
O2	0.0419 (19)	0.0602 (19)	0.090 (2)	−0.0187 (17)	0.0097 (17)	−0.0099 (17)
O3	0.0493 (19)	0.0521 (16)	0.0664 (18)	−0.0012 (17)	0.0193 (17)	−0.0176 (16)
O4	0.0379 (17)	0.0378 (16)	0.0616 (17)	0.0044 (15)	−0.0089 (15)	0.0006 (14)
O5	0.070 (2)	0.0578 (17)	0.0451 (15)	0.0006 (18)	−0.0025 (17)	−0.0202 (14)
O6	0.0498 (17)	0.0501 (15)	0.0450 (14)	0.0078 (15)	−0.0096 (16)	0.0026 (14)
O7	0.0423 (19)	0.0594 (19)	0.0515 (17)	−0.0092 (17)	−0.0123 (15)	0.0064 (15)
C1	0.038 (2)	0.035 (2)	0.038 (2)	−0.005 (2)	0.0047 (19)	0.0021 (19)
C2	0.026 (2)	0.0382 (19)	0.0401 (19)	0.0016 (18)	0.0017 (18)	0.0050 (17)
C3	0.038 (2)	0.037 (2)	0.038 (2)	0.000 (2)	0.002 (2)	−0.0021 (18)
C4	0.033 (2)	0.038 (2)	0.044 (2)	−0.004 (2)	0.0014 (19)	0.0091 (19)
C5	0.043 (3)	0.054 (2)	0.056 (2)	0.005 (2)	0.021 (2)	0.012 (2)
C6	0.054 (3)	0.049 (2)	0.061 (3)	−0.009 (3)	−0.001 (3)	−0.009 (2)
C7	0.024 (2)	0.038 (2)	0.050 (2)	−0.0038 (19)	−0.0010 (19)	−0.002 (2)
C8	0.025 (2)	0.0350 (19)	0.0419 (19)	−0.0014 (18)	−0.0005 (19)	0.0004 (16)
C9	0.029 (2)	0.039 (2)	0.040 (2)	−0.0013 (19)	−0.0042 (19)	0.0038 (18)
C10	0.037 (2)	0.042 (2)	0.049 (2)	−0.006 (2)	0.003 (2)	0.002 (2)
C11	0.060 (3)	0.059 (3)	0.054 (3)	−0.002 (3)	−0.028 (2)	0.002 (2)
C12	0.064 (3)	0.037 (2)	0.070 (3)	0.011 (2)	−0.003 (3)	−0.005 (2)
C13	0.029 (2)	0.0339 (17)	0.044 (2)	−0.0009 (18)	0.0045 (19)	−0.0019 (17)
C14	0.046 (3)	0.039 (2)	0.036 (2)	−0.006 (2)	0.000 (2)	−0.0030 (17)
C15	0.064 (3)	0.054 (2)	0.045 (2)	−0.016 (3)	0.004 (2)	0.001 (2)

### Geometric parameters (Å, º)

**Table:**

N1—C1	1.354 (4)	C5—H5A	0.9700
N1—C4	1.400 (4)	C5—H5B	0.9700
N1—C5	1.475 (4)	C5—H5C	0.9700
N2—C3	1.371 (4)	C6—H6A	0.9700
N2—C4	1.373 (4)	C6—H6B	0.9700
N2—C6	1.476 (4)	C6—H6C	0.9700
N3—C7	1.377 (4)	C7—C8	1.507 (4)
N3—C10	1.384 (4)	C8—C9	1.504 (4)
N3—C11	1.469 (4)	C8—C13	1.543 (4)
N4—C9	1.368 (4)	C8—H8	0.9900
N4—C10	1.381 (4)	C11—H11A	0.9700
N4—C12	1.466 (4)	C11—H11B	0.9700
O1—C1	1.224 (4)	C11—H11C	0.9700
O2—C4	1.211 (4)	C12—H12A	0.9700
O3—C3	1.206 (4)	C12—H12B	0.9700
O4—C7	1.208 (4)	C12—H12C	0.9700
O5—C10	1.199 (4)	C13—C14	1.525 (4)
O6—C9	1.207 (4)	C13—H13	0.9900
O7—C14	1.209 (4)	C14—C15	1.496 (5)
C1—C2	1.489 (4)	C15—H15A	0.9700
C2—C3	1.498 (4)	C15—H15B	0.9700
C2—C13	1.537 (4)	C15—H15C	0.9700
C2—H2	0.9900		
			
C1—N1—C4	124.7 (3)	N3—C7—C8	116.2 (3)
C1—N1—C5	118.2 (3)	C9—C8—C7	116.1 (3)
C4—N1—C5	117.0 (3)	C9—C8—C13	114.6 (3)
C3—N2—C4	124.1 (3)	C7—C8—C13	112.6 (3)
C3—N2—C6	118.3 (3)	C9—C8—H8	103.9
C4—N2—C6	117.6 (3)	C7—C8—H8	103.9
C7—N3—C10	125.4 (3)	C13—C8—H8	103.9
C7—N3—C11	119.0 (3)	O6—C9—N4	122.1 (3)
C10—N3—C11	115.7 (3)	O6—C9—C8	121.2 (3)
C9—N4—C10	125.1 (3)	N4—C9—C8	116.4 (3)
C9—N4—C12	118.9 (3)	O5—C10—N4	121.7 (3)
C10—N4—C12	115.9 (3)	O5—C10—N3	120.8 (3)
O1—C1—N1	121.1 (3)	N4—C10—N3	117.5 (3)
O1—C1—C2	121.0 (3)	N3—C11—H11A	109.5
N1—C1—C2	117.8 (3)	N3—C11—H11B	109.5
C1—C2—C3	116.1 (3)	H11A—C11—H11B	109.5
C1—C2—C13	115.3 (2)	N3—C11—H11C	109.5
C3—C2—C13	109.1 (2)	H11A—C11—H11C	109.5
C1—C2—H2	105.0	H11B—C11—H11C	109.5
C3—C2—H2	105.0	N4—C12—H12A	109.5
C13—C2—H2	105.0	N4—C12—H12B	109.5
O3—C3—N2	119.9 (3)	H12A—C12—H12B	109.5
O3—C3—C2	122.2 (3)	N4—C12—H12C	109.5
N2—C3—C2	117.8 (3)	H12A—C12—H12C	109.5
O2—C4—N2	121.8 (3)	H12B—C12—H12C	109.5
O2—C4—N1	120.4 (3)	C14—C13—C2	110.4 (3)
N2—C4—N1	117.9 (3)	C14—C13—C8	113.0 (3)
N1—C5—H5A	109.5	C2—C13—C8	116.2 (2)
N1—C5—H5B	109.5	C14—C13—H13	105.4
H5A—C5—H5B	109.5	C2—C13—H13	105.4
N1—C5—H5C	109.5	C8—C13—H13	105.4
H5A—C5—H5C	109.5	O7—C14—C15	122.5 (3)
H5B—C5—H5C	109.5	O7—C14—C13	119.9 (3)
N2—C6—H6A	109.5	C15—C14—C13	117.1 (3)
N2—C6—H6B	109.5	C14—C15—H15A	109.5
H6A—C6—H6B	109.5	C14—C15—H15B	109.5
N2—C6—H6C	109.5	H15A—C15—H15B	109.5
H6A—C6—H6C	109.5	C14—C15—H15C	109.5
H6B—C6—H6C	109.5	H15A—C15—H15C	109.5
O4—C7—N3	122.2 (3)	H15B—C15—H15C	109.5
O4—C7—C8	121.5 (3)		
			
C4—N1—C1—O1	178.9 (3)	O4—C7—C8—C13	30.0 (4)
C5—N1—C1—O1	−5.1 (5)	N3—C7—C8—C13	−152.3 (3)
C4—N1—C1—C2	−4.5 (4)	C10—N4—C9—O6	172.4 (3)
C5—N1—C1—C2	171.5 (3)	C12—N4—C9—O6	−6.2 (5)
O1—C1—C2—C3	−170.6 (3)	C10—N4—C9—C8	−14.2 (5)
N1—C1—C2—C3	12.8 (4)	C12—N4—C9—C8	167.2 (3)
O1—C1—C2—C13	−41.1 (4)	C7—C8—C9—O6	−165.3 (3)
N1—C1—C2—C13	142.3 (3)	C13—C8—C9—O6	−31.3 (4)
C4—N2—C3—O3	−174.4 (3)	C7—C8—C9—N4	21.3 (4)
C6—N2—C3—O3	3.9 (4)	C13—C8—C9—N4	155.2 (3)
C4—N2—C3—C2	8.0 (4)	C9—N4—C10—O5	−176.3 (3)
C6—N2—C3—C2	−173.7 (3)	C12—N4—C10—O5	2.3 (5)
C1—C2—C3—O3	168.0 (3)	C9—N4—C10—N3	2.2 (5)
C13—C2—C3—O3	35.6 (4)	C12—N4—C10—N3	−179.2 (3)
C1—C2—C3—N2	−14.5 (4)	C7—N3—C10—O5	−179.2 (3)
C13—C2—C3—N2	−146.9 (3)	C11—N3—C10—O5	1.1 (5)
C3—N2—C4—O2	179.9 (3)	C7—N3—C10—N4	2.3 (5)
C6—N2—C4—O2	1.6 (5)	C11—N3—C10—N4	−177.3 (3)
C3—N2—C4—N1	0.8 (4)	C1—C2—C13—C14	−75.7 (3)
C6—N2—C4—N1	−177.5 (3)	C3—C2—C13—C14	57.1 (3)
C1—N1—C4—O2	178.2 (3)	C1—C2—C13—C8	54.7 (4)
C5—N1—C4—O2	2.2 (4)	C3—C2—C13—C8	−172.4 (3)
C1—N1—C4—N2	−2.8 (4)	C9—C8—C13—C14	40.2 (4)
C5—N1—C4—N2	−178.7 (3)	C7—C8—C13—C14	175.8 (3)
C10—N3—C7—O4	−176.3 (3)	C9—C8—C13—C2	−89.0 (3)
C11—N3—C7—O4	3.3 (5)	C7—C8—C13—C2	46.6 (4)
C10—N3—C7—C8	5.9 (4)	C2—C13—C14—O7	11.0 (4)
C11—N3—C7—C8	−174.4 (3)	C8—C13—C14—O7	−121.2 (3)
O4—C7—C8—C9	164.9 (3)	C2—C13—C14—C15	−161.4 (3)
N3—C7—C8—C9	−17.4 (4)	C8—C13—C14—C15	66.5 (4)
